# Water Color Identification System for Monitoring Aquaculture Farms

**DOI:** 10.3390/s22197131

**Published:** 2022-09-20

**Authors:** Hsiang-Chieh Chen, Sheng-Yao Xu, Kai-Han Deng

**Affiliations:** Department of Electrical Engineering, National United University, Miaoli 360302, Taiwan

**Keywords:** aquaculture, color correction, color identification, image segmentation, smart agriculture, water color

## Abstract

This study presents a vision-based water color identification system designed for monitoring aquaculture ponds. The algorithm proposed in this system can identify water color, which is an important factor in aquaculture farming management. To address the effect of outdoor lighting conditions on the proposed system, a color correction method using a color checkerboard was introduced. Several candidates for water-only image patches were extracted by performing image segmentation and fuzzy inferencing. Finally, a deep learning-based model was employed to identify the color of these patches and then find the representative color of the water. Experiments at different aquaculture sites verified the effectiveness of the proposed system and its algorithm. The color identification accuracy exceeded 96% for the test data.

## 1. Introduction

Aquaculture plays an important role in fisheries and feeds large populations of people worldwide. According to a long-term report by the Food and Agriculture Organization, marine fish resources are depleting [1]. Therefore, government institutions and private organizations have implemented many interventions to raise awareness of the importance of global fishery resources. Fishing regulations and ocean environment conservation are helpful for striking a balance between food and sustainability. The production of captured fisheries has stagnated since the 1990s, and aquaculture production appears to be the solution to fill this shortage in consumption requirements [2]. However, aquaculture production must also continuously increase to meet the demand for food given the growing population. Many public and private initiatives have started to intensify or develop technologies to increase aquaculture production [3,4,5]. The productivity and efficiency of aquaculture farms depend on various factors such as technology development and application, geophysical situations, and the market and social conditions under which the farms are managed [2,6]. To maintain a suitable aquatic environment for fish and organisms, farmers usually observe the water color and the presence of phytoplankton in the pond for management purposes.

Vision-based monitoring techniques have become widely used in many fields, including aquaculture, as computer vision technology has matured [7]. Currently, the utilization of computer vision mainly focuses on different aspects of recognition [8], such as counting [9,10,11], size measurement [12,13], weight estimation [14,15,16], gender identification [17,18], and species identification [19,20]. In addition to monitoring aquarium organisms, status monitoring of farming environments is equally important. The productivity and success rate of breeding are highly related to water quality. Several factors are critical for the survival and growth of cultured species [8], for instance, temperature, pH, dissolved oxygen, nitrite, nitrate, etc. They can be measured by specified sensors. In addition to the above factors, water color is also a factor worthy of observation [21]. For an experienced farmer, water color is a subjective indicator that can represent water quality and can be directly observed by human eyes when no water quality sensing system is built. In surveyed investigations, water color often varies with time and location [2]. For example, it became muddy (represented by a dark green color) after feeding, which is always preferable for aquaculture species. Mane et al. [22] found that water with phytoplankton (represented by a light green color) was highly productive, whereas clear water was less productive. The authors of [23] reported that farmers with muddy or green-colored water gained more productivity than farmers with blue-green water in their ponds. Field observations also suggest that controlling the amount of phytoplankton in the water, with neither too little nor too much phytoplankton, is useful for efficiently increasing production. The above statements show the importance of water color in the aquaculture breeding process. Therefore, the goal of this study was to introduce a vision-based water color monitoring system and its algorithm. The proposed algorithm can identify 19 categories of water colors that match the aquatic product production and sales resume system provided by the Fisheries Agency, Council of Agriculture (FA-COA), Taiwan. Certainly, the definitions of these colors can be changed according to the regulations of different regions and countries.

The water identification of such a vision system is generally divided into several stages, including the steps of (1) restoring the color of the captured image as close to the human eye sees as possible; (2) extracting the water area within the image; and (3) recognizing the color of the water area and obtaining the representative water color of the aquaculture farm. For the color issue, the majority of methods considered color correction problems as finding the transformation between captured and ideal colors. In [24], color homographies were applied to color correction, and the results showed that colors across a change in viewing conditions were related to homographies. This article gave us a good idea of using color-reference objects, namely color checkers and checkerboards. Nomura et al. [25] also used color checkerboards to restore underwater color. Second, semantic segmentation was extensively applied to extract desired regions, such as the water region in this study. In recent years, many studies have presented deep learning-based methods for image segmentation tasks. Furthermore, several review articles have been published to compare commonly used methods [26,27,28,29,30]. The authors of [26] categorized methods based on the degree of supervision during the training process and focused on real-time segmentation. The author of [27] mainly described classical learning-based methods such as support vector machines and decision trees. A detailed introduction and comprehensive comparison were provided in [28,29], including network architectures, datasets, and metrics in the field of semantic segmentation. For the needs of this study, the segmentation result only draws the approximate regions occupied by water. In this study, the YOLACT-based method [31,32] with our modifications was selected for use after evaluating the accuracy and time efficiency. Finally, the color identification method is relatively simple because a reliable water region is extracted. Deep architectures, such as convolutional neural networks (CNNs), have verified their superiority over other existing methods. They are currently the most popular approach for classification tasks. CNN-based models can be trained using end-to-end learning without designating task-related feature extractors. The VGG-16 and VGG-19 models proposed in [33] are extremely popular and significantly improve the AlexNet [34] by enlarging the filters and adding more convolution layers. However, deeper neural networks are often more difficult to train. He et al. [35] presented a residual learning framework to simplify the training of deep networks. Their proposed residual networks (ResNets) are easy to optimize and can obtain a high level of accuracy from the remarkably increased depth of the network.

Based on the above investigations, we designed an algorithm suitable for our proposed system that can identify water color in an aquaculture pond. The main contributions of this study are summarized as follows:

1. We designed a color checkerboard based on 24 colors commonly used for color correction. We then adopted this checkerboard to correct the colors of images captured under various lighting conditions in the outside environment.
2. We proposed a scheme for extracting candidate patches from the water regions in an image. These candidate patches are further used to identify the representative color of a pond. The proposed scheme consists of two main steps: semantic segmentation and fuzzy inferencing to determine the degree to which a specified image patch is considered to be the candidate patch.
3. A simple color identification model with a deep CNN was implemented. The model’s output is the probability of belonging to one of the predefined color categories.

The remainder of this paper is organized as follows. Section 2 introduces the proposed system and its main algorithm for achieving the water color identification of an aquaculture pond. The implementation details and experimental results are presented in Section 3. Section 4 provides additional discussions on the proposed system. Finally, the conclusions are presented in Section 5. 

## 2. The Proposed System and Algorithm

### 2.1. System Overview

**Figure 1:**
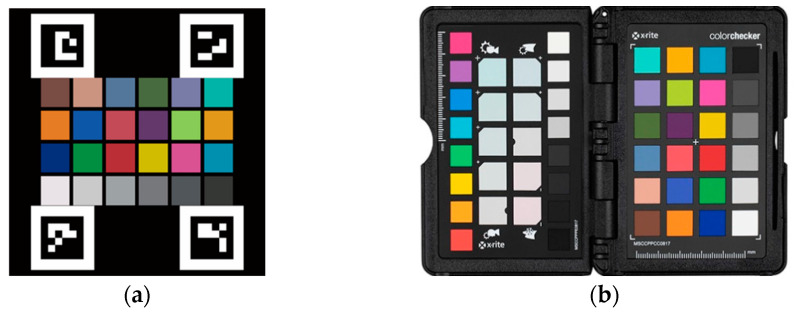
Our own designed color checkerboard (**a**), and the referenced ColorChecker Passport (**b**).

**Figure 2:**
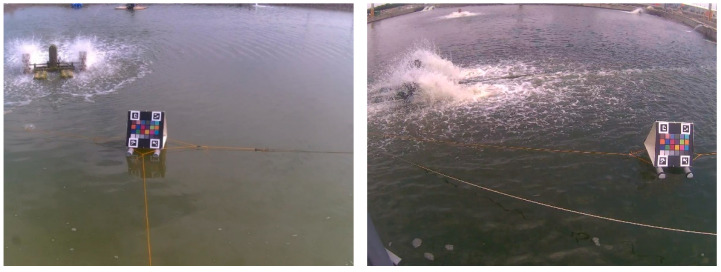
Two real scenes in which the color checkerboard is placed.

**Figure 3:**
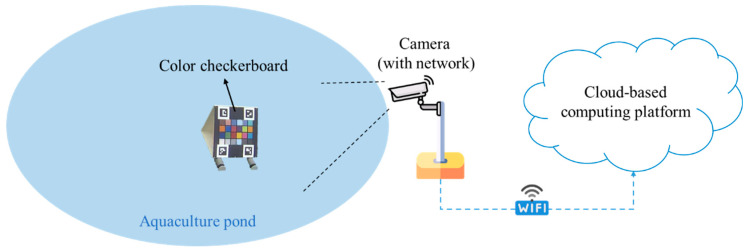
The proposed system established at the experimental site.

The proposed water color monitoring system consists of three main components: (1) a color camera fixed by the pond; (2) a color checkerboard placed on the water; and (3) a cloud-based computing platform where the proposed algorithm is deployed. The aforementioned color checkerboard with 24 color blocks, as illustrated in Figure 1, is our own design, inspired by the x-rite ColorChecker Passport Photo 2 [36]. In addition, there are four ArUco markers [37] on the corners of the checkerboard. These four markers were helpful for the localization of the 24 color blocks. Table 1 lists the RGB information of the 24 colors defined in this study. The purpose of using this color checkerboard is to correct the image color back to a color that is close to what the human eye sees. Figure 2 shows two image shots of real scenes in which color checkerboards were placed. An overview of the proposed system established at each experimental site is shown in Figure 3.

### 2.2. Main Algorithm of Water Color Identification

In this subsection, we describe the main procedure of our proposed water color identification algorithm, which includes four stages: (1) image color correction, (2) image segmentation, (3) candidate patch extraction, and (4) color identification of candidate patches.

#### 2.2.1. Image Color Correction

**Figure 4:**
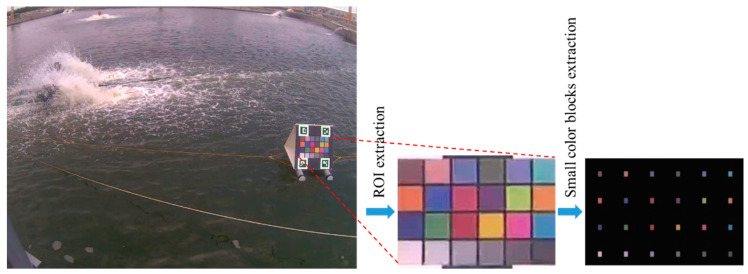
Results of ArUco marker detection, and ROI and small color blocks extraction.

Owing to the diverse changes in outdoor lighting conditions, errors may occur while identifying the water color unless the image color can be corrected. To make the correction easy and stable, we used a color checkerboard to correct the color information of the captured image. Because the information of 24 color blocks on this checkerboard is known, a transformation for restoring the colors to their ideal values for capture can be found. Thus, the color of each image shot was corrected using this transformation. In our method, the checkerboard is first localized using the four ArUco markers. Given an image containing ArUco markers, the position and ID number of each marker can be easily obtained using the algorithm in [38]. Furthermore, the popular open-source library OpenCV provides an ArUco module for generating and detecting these markers [37]. From the relative positions of the four markers, the region of interest (ROI) was extracted and then normalized to a predefined size. Consequently, 24 smaller color blocks were obtained simply from this normalized ROI because the layout of the checkerboard was designed by us. Figure 4 shows the results of the extraction of the ROI and its 24 small color blocks.

Let ${\left[R^{i},G^{i},B^{i}\right]}^{T}$ be the mean RGB value of all pixels inside the i-th small color block, where i=1,2,…,24. It is first converted into the CIE Lab color model via the following transformation formulae:

(1) $$\left[\begin{array}{c} X^{i}\\ Y^{i}\\ Z^{i} \end{array}\right]=\left[\begin{array}{ccc} 0.412453 & 0.357580 & 0.180423\\ 0.212671 & 0.715160 & 0.072169\\ 0.019334 & 0.119193 & 0.950227 \end{array}\right]\left[\begin{array}{c} R^{i}\\ G^{i}\\ B^{i} \end{array}\right]$$

(2) $$\left[\begin{array}{c} L^{i}\\ a^{i}\\ b^{i} \end{array}\right]=\left[\begin{array}{c} 116f\left(\frac{Y^{i}}{Y_{n}}\right)\\ 500\left(f\left(\frac{X^{i}}{X_{n}}\right)-f\left(\frac{Y^{i}}{Y_{n}}\right)\right)\\ 200\left(f\left(\frac{Y^{i}}{Y_{n}}\right)-f\left(\frac{Z^{i}}{Z_{n}}\right)\right) \end{array}\right]$$

where $X_{n}=0.9515$, $Y_{n}=1.0$, $Z_{n}=1.0886$, and

(3) $$f\left(v\right)=\{\begin{array}{cc} v^{\frac{1}{3}}, & if\;v>0.008856;\\ 7.787037v+\frac{16}{116}, & \mathrm{otherwise}. \end{array}$$

For an image captured at timestamp t, there are 24 vectors ${\left[L_{t}^{i},a_{t}^{i},b_{t}^{i}\right]}^{T}$ for 1≤i≤24 representing 24 color blocks. Let ${\left[L_{GT}^{i},a_{GT}^{i},b_{GT}^{i}\right]}^{T}$ be the ground truth of the i-th color block, which can be derived from the RGB values listed in Table 1, and (1)–(3). We assume that there exists a transformation matrix M denoted by:

(4) $$M=\left[\begin{array}{ccc} m_{11} & m_{12} & m_{13}\\ m_{21} & m_{22} & m_{23}\\ m_{31} & m_{32} & m_{33} \end{array}\right]$$

which can be applied to correct the real-world captured ${\left[L_{t}^{i},a_{t}^{i},b_{t}^{i}\right]}^{T}$ back to the ground truth ${\left[L_{GT}^{i},a_{GT}^{i},b_{GT}^{i}\right]}^{T}$. Consequently, the matrix M can be estimated using the least-squares error method as follows:

(5) $${\left[\begin{array}{ccccccccc} L_{t}^{1} & a_{t}^{1} & b_{t}^{1} & 0 & 0 & 0 & 0 & 0 & 0\\ 0 & 0 & 0 & L_{t}^{1} & a_{t}^{1} & b_{t}^{1} & 0 & 0 & 0\\ 0 & 0 & 0 & 0 & 0 & 0 & L_{t}^{1} & a_{t}^{1} & b_{t}^{1}\\ \vdots & \vdots & \vdots & \vdots & \vdots & \vdots & \vdots & \vdots & \vdots \\ L_{t}^{24} & a_{t}^{24} & b_{t}^{24} & 0 & 0 & 0 & 0 & 0 & 0\\ 0 & 0 & 0 & L_{t}^{24} & a_{t}^{24} & b_{t}^{24} & 0 & 0 & 0\\ 0 & 0 & 0 & 0 & 0 & 0 & L_{t}^{24} & a_{t}^{24} & b_{t}^{24} \end{array}\right]}_{72\times 9}{\left[\begin{array}{c} m_{11}\\ m_{12}\\ m_{13}\\ m_{21}\\ m_{22}\\ m_{23}\\ m_{31}\\ m_{32}\\ m_{33} \end{array}\right]}_{9\times 1}={\left[\begin{array}{c} L_{GT}^{1}\\ a_{GT}^{1}\\ b_{GT}^{1}\\ \vdots \\ L_{GT}^{24}\\ a_{GT}^{24}\\ b_{GT}^{24} \end{array}\right]}_{72\times 1}$$

Equation (5) can be abbreviated as $A_{72\times 9}m_{9\times 1}=d_{9\times 1}$. Vector m that is reshaped from M is solved using a pseudo-inverse.

(6) $$m={\left(A^{T}A\right)}^{-1}A^{T}d$$

**Figure 5:**
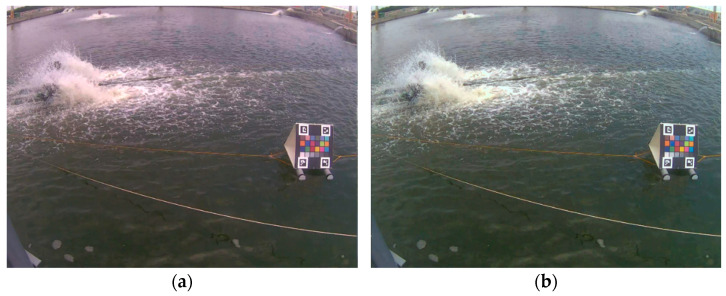
The images of a real scene: (**a**) original capture, and (**b**) after color correction.

Therefore, the color correction of an image can be implemented, by the steps of pseudocode summarized in the following Algorithm 1. Figure 5 shows the results before and after color correction for a real scene. The image obtained after performing color correction is closer to that of the human eye.
**Algorithm 1:** Proposed color correction algorithm (Pseudocode)Input: an image capture (I), with the size of image width (W) and image height (H)Output: an image after color correction1:p[1]←(0,0), p[2]←(0,0), p[3]←(0,0), p[4]←(0,0) //Initialize four marker positions2:dict←cv2.aruco.Dictionary_get(cv2.aruco.DICT_4×4_50) //Initialize ArUco marker dictionary3:p[i],ids[i]←cv2.aruco.detectMarkers(I,dict) //Detect ArUco markers, return their positions and IDs by OpenCV4:h←cv2.findHomography(p, c) //Find transformation matrix (geometric homography) by OpenCV5:$I_{n}\leftarrow \mathrm{cv}2.\mathrm{warpPerspective}\left(I,h,200,200\right)$ //Normalize the ROI into the size 200×200 pixels by OpenCV6:Detect 24 color blocks in normalized ROI //Use OpenCV APIs to detect color blocks7:**for** each color block i in 1 to 24 **do**:8: Calculate its mean RGB value: $R^{i}$, $G^{i}$, $B^{i}$9: Convert to Lab value from RGB: $L^{i}$, $a^{i}$, $b^{i}$ through Equations (1)–(3).10:**end for**11:Calculate transformation matrix M through Equations (4) and (6).12:**for** every pixel (x,y) in I **do**:13: Convert its RGB value ${\left[R_{\left(x,y\right)},G_{\left(x,y\right)},B_{\left(x,y\right)}\right]}^{T}$ to ${\left[L_{\left(x,y\right)},a_{\left(x,y\right)},b_{\left(x,y\right)}\right]}^{T}$14: Compute the color-corrected value as ${\left[L_{\left(x,y\right)}^{\prime },a_{\left(x,y\right)}^{\prime },b_{\left(x,y\right)}^{\prime }\right]}^{T}=M{\left[L_{\left(x,y\right)},a_{\left(x,y\right)},b_{\left(x,y\right)}\right]}^{T}$15: Transform the corrected Lab value back to RGB color model.16:**end for**

#### 2.2.2. Image Segmentation and Candidate Patch Extraction

Before water color identification, it is critical to obtain an image patch that covers only the water surface. For a simpler description, we consider the image on the right in Figure 5 as an example for describing the proposed method. It can be observed that there are some undesired objects, such as waterwheels and foams, that should be excluded during the process of water color identification. Therefore, in this subsection, we introduce a method for cropping several small patches that cover only the water surface. Our proposed method comprises two main stages: segmentation of the water region and extraction of water-only image patches.

- Stage 1: Segmentation of water region.

**Figure 6:**
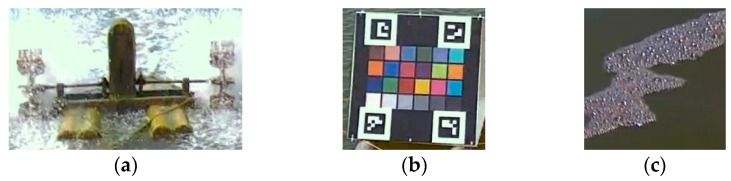
Three classes must be excluded: (**a**) waterwheel, (**b**) checkerboard, and (**c**) foams.

**Figure 7:**
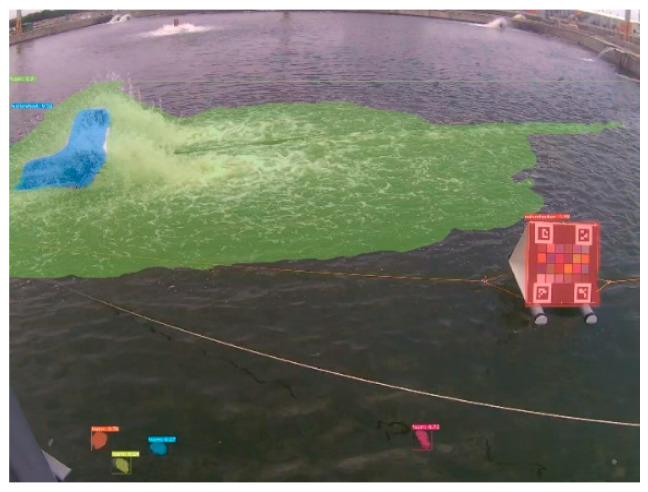
Result of YOLACT-based instance segmentation.

Generally, several types of objects may appear in aquaculture ponds. In this study, we first implemented a pixel-level segmentation method based on the improved YOLACT architecture [32]. Here, the implemented YOLACT-based model was further modified. To obtain precise segmentation results for a large capture, the backbone of this model was replaced by a ResNet-200 [39], whose input achieved 700 × 700 pixels. Except for the water region, we additionally define three classes: the waterwheel, color checkerboard, and foams on water, which are further excluded while performing water segmentation. Certainly, these classes can be modified according to the needs of different aquaculture farms. Figure 6 shows examples of these three classes, and Figure 7 shows the results of the implemented segmentation method. For ease of observation, the water region is not illustrated. Therefore, the candidates of water patches used for color identification can be extracted from the segmented water regions without other undesired objects.

- Stage 2: Extraction of candidates from image patches.

**Figure 8:**
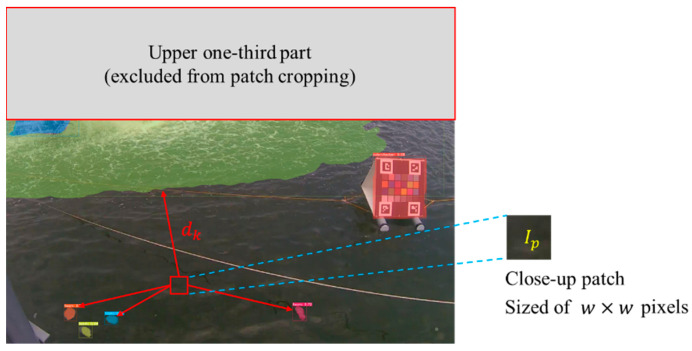
Close-up of an image patch and distances from foams.

This step extracts a certain number of candidate image patches, which are classified in terms of their colors, from the water regions. According to our observational experience at many experimental sites, the distant part of the image is often affected by light reflection and refraction. Therefore, we prevented cropping the water patch in the upper one-third of the image. Let $I_{p}$ denote an image patch with the size of w×w pixels; its position is generated randomly within the lower two-thirds of an image, as shown in Figure 8. Assuming that there are K groups of foams and $d_{k}$ represents the minimum distance from the patch $I_{p}$ to the k-th foam contour, for k=1,2,…,K. Every $d_{k}$ can be easily calculated by the well-known connected component labeling and image geometry techniques. Next, we define the intensity variable as the standard deviation σ of the intensities of all the pixels within the patch, formulated as follows:

(7) $$\sigma =\sqrt{\frac{1}{w\times w}\underset{\left(x,y\right)\in I_{p}}{\sum }f{\left(x,y\right)}^{2}-\mu^{2}}$$

(8) $$\mu =\frac{1}{w\times w}\underset{\left(x,y\right)\in I_{p}}{\sum }f\left(x,y\right)$$

where $\left(x,y\right)\in I_{p}$ denotes every pixel in the patch, which is converted into grayscale with the pixel values f(x,y). A small intensity deviation implies that the patch is flat (or called textureless). The selection criterion for whether a specified patch is selected as a candidate for color identification is presented below.

*“The farther the patch is distant from the foams and the flatter the patch texture is, the higher the probability that the patch will be selected as a candidate”.*

**Figure 9:**
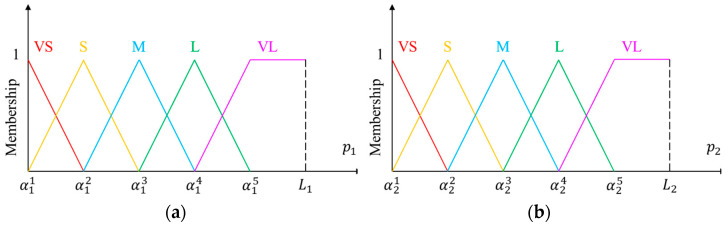
Fuzzy sets of antecedent variables: (**a**) input 1, and (**b**) input 2.

**Figure 10:**
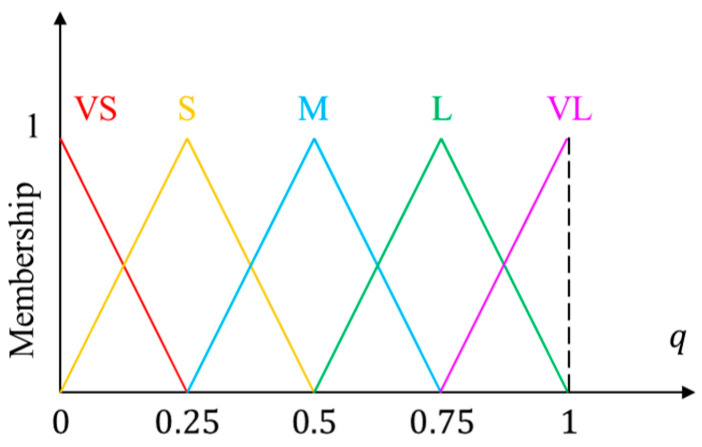
Fuzzy sets of consequent variables.

In this study, a fuzzy inference system (FIS) is proposed to determine the above probability. Let $p_{1}=\underset{1\leq k\leq K}{\min}d_{k}$ and $p_{2}=\sigma$ be the two antecedent variables of our proposed FIS, and q be its consequent variable. Here, $p_{1}$ represents the minimal distance between the patch and all foams, and $p_{2}$ implicitly expresses the flatness of the patch. Assuming that $p_{1}$ ranges in $\left[0,L_{1}\right]$ and $p_{2}$ ranges in $\left[0,L_{2}\right]$, where $L_{1}$ is the length of the diagonal of the lower two-thirds of the image and $L_{2}=255$ for gray images. The fuzzy sets of the antecedent variables are depicted in Figure 9, in which triangular and trapezoidal functions are selected as the membership functions. For the consequent variable, q is represented by equally spaced triangular membership functions, as shown in Figure 10. The linguistic terms in Figs. 9 and 10 include: very small (VS), small (S), medium (M), large (L), and very large (VL). The parameters for defining the five fuzzy sets of antecedent variables were $\left\{\alpha_{1}^{n}\;|\;n=1,2,\ldots ,5\right\}$ and $\left\{\alpha_{2}^{n}\;|\;n=1,2,\ldots ,5\right\}$. For simplicity, we only attempted to determine the values of $\alpha_{1}^{5}$ and $\alpha_{2}^{5}$, and set $\alpha_{1}^{1}=\alpha_{2}^{1}=0$; meanwhile, the other parameters were equally spaced between them. Table 2 lists the parameters determined by the trial-and-error method used in this study.

According to the aforementioned selection criterion for a water-only image patch, we used the variables $p_{1}$ and $p_{2}$ as two inputs in the proposed FIS and objectively constructed fuzzy rules using the above linguistic terms. For example:

$$\mathrm{IF}\;p_{1}\;\mathrm{is}\;\mathrm{large}\;(L)\;\mathrm{AND}\;p_{2}\;\mathrm{is}\;\mathrm{small}\;(S),\;\mathrm{THEN}\;q\;\mathrm{is}\;\mathrm{large}\;(L).$$

The consequent variable q indicates the degree to which a specified crop is considered a water-only patch for the subsequent color identification process. All 25 fuzzy rules are listed in Table 3. The r-th fuzzy rule can be formally written in the following format.

$$\mathrm{Rule}\;r:\;\mathrm{IF}\;p_{1}\;\mathrm{is}\;{\hat{A}}_{1}^{r}\;\mathrm{AND}\;p_{2}\;\mathrm{is}\;{\hat{A}}_{2}^{r},\;\mathrm{THEN}\;q\;\mathrm{is}\;{\hat{B}}^{r}.$$

Here, r=1,2,…,25, and ${\hat{A}}_{1}^{r}\in \left\{\mathrm{VS},S,M,L,\mathrm{VL}\right\}$, ${\hat{A}}_{2}^{r}\in \left\{\mathrm{VS},S,M,L,\mathrm{VL}\right\}$, and ${\hat{B}}^{r}\in \left\{\mathrm{VS},S,M,L,\mathrm{VL}\right\}$ were selected from the fuzzy sets of $p_{1}$, $p_{2}$, and q, respectively. Whereas an input pair $\left({\tilde{p}}_{1},{\tilde{p}}_{2}\right)$ enters the FIS and fires some fuzzy rules, the crisp output derived by the proposed FIS is obtained using the minimum inference engine and center-of-gravity defuzzification method [40], as follows:

(9) $$\tilde{q}=\frac{\int_{Q}^{}q\cdot B^{\prime }\left(q\right)dq}{\int_{Q}^{}B^{\prime }\left(q\right)dq}$$

and

(10) $$B^{\prime }\left(q\right)=\underset{r}{\max}\left\{{\hat{A}}_{1}^{r}\left({\tilde{p}}_{1}\right)\wedge {\hat{A}}_{2}^{r}\left({\tilde{p}}_{2}\right)\wedge {\hat{B}}^{r}\left(q\right)\right\}$$

where ∧ is the minimum operator and Q is the universe of discourse of the consequent variable. Therefore, the derived degree $\tilde{q}$ is proportional to its selection as a candidate for any specified image patch. The steps for extracting a certain number of candidate patches can be performed using the following Algorithm 2, which is summarized by pseudocode.
**Algorithm 2:** Proposed water-only candidate extraction algorithm (pseudocode)Input: a color-corrected image ($I_{c}$), with the size of image width (W) and image height (H)Output: N candidates for water-only patches.1:N←11, w←W/20 //Initialize parameters2:n←0 //Initialize a counter3:T←0.5 //Initialize the threshold4:**while** n<N **do**:5: x←randint(w/2,W−w/2), y←randint(H/3+w/2,H−w/2) //Randomly generate pixel (x,y) in lower 2/3 part6:$\;x_{1}\leftarrow x-w/2$, $y_{1}\leftarrow y-w/2$, $x_{2}\leftarrow x+w/2$, $y_{2}\leftarrow y+w/2$ //Determine the coordinates of corners7: Crop the patch $I_{p}$, whose upper-left corner is $\left(x_{1},y_{1}\right)$ and the lower-right corner is $\left(x_{2},y_{2}\right)$8: **for** k in 1 to K **do**:9:$\;\;{\tilde{p}}_{1}\leftarrow \min\left(d_{k},p_{1}\right)$ //Set the minimum of $d_{k}$s to be $p_{1}$10: **end for**
11: Calculate ${\tilde{p}}_{2}$ through Equations (7) and (8)12: Import the input pair $\left({\tilde{p}}_{1},{\tilde{p}}_{2}\right)$ into the proposed FIS13: Derive the crisp output $\tilde{q}$ of the FIS14: **if** $\tilde{q}>T$ **then do**:15:$\;\;\mathrm{Candidate}\;\mathrm{patch}\leftarrow I_{p}$16:   *n* ← *n* + 117: **end if**
18:**end while**

#### 2.2.3. Color Identification

In this subsection, a learning-based color identification method is applied to each of the candidate patches. Thus, most of the identified results can be regarded as the representative water color of the aquaculture pond. The classes of water color identification were divided into six major colors, including green, brown, red, yellow, dark, and blue, indexed in order from 001 to 006. They can be further subdivided into 19 types. Table 4 lists the codes of these water colors, which match the aquatic product production and sales resume system provided by the FA-COA, Taiwan. In addition, there is another color type known as the unknown class. These color codes can vary according to national regulations.

**Figure 11:**
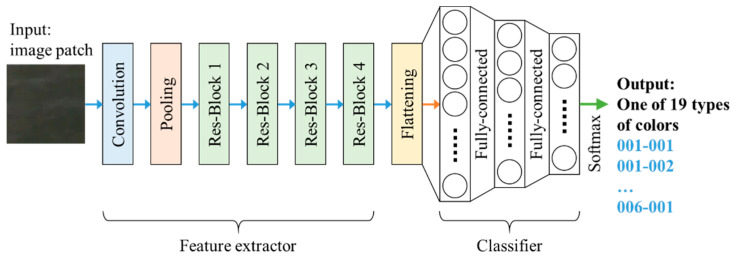
Deep CNN with ResNet-50 backbone for water color identification.

To perform the task of color identification, a deep learning-based model with parts of feature extraction and classification was used in this study. Numerous backbones with deep architectures have verified the effectiveness of feature extraction. During the pre-research stage, we evaluated several popular feature extraction backbones, including VGG-16, VGG-19 [33], ResNet-50 [35], InceptionV3 [41], and MobileNet [42]. ResNet-50 was finally selected as the image extractor of our proposed model because it performed well in feature extraction in the experiments. This image extractor was followed by a fully connected network designed to perform classification. Figure 11 illustrates the architecture of the proposed method for color identification. The details of its implementation and performance evaluation are described in Section 3.3.

## 3. Implementation and Experimental Results

First, we selected several aquaculture farms in Taiwan to implement the proposed system. There were a total of eight experimental sites for the verification of the proposed technology. All images in our experiments were actual shots of the scenes. The system at every experimental site consisted of a camera, a checkerboard, and a cloud-based computing platform. In this section, we focus on the results of (1) color correction, (2) image segmentation, and (3) water color identification. GPU-accelerated techniques were used to implement the proposed method to satisfy the computational requirements of running a deep learning-based model. The algorithm was programmed using Python language.

### 3.1. Results of Color Correction

**Figure 12:**
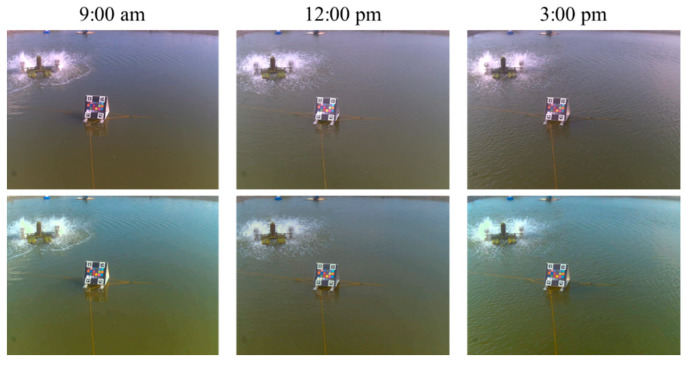
Results of color correction on site: original images (**upper**), and the color-corrected images (**bottom**).

**Figure 13:**
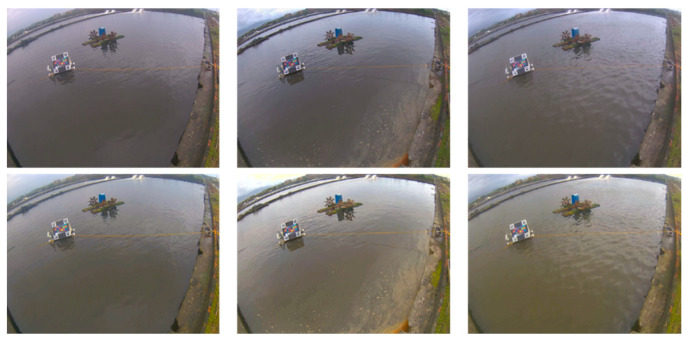
More results of color correction on site: original images (**upper**), and the color-corrected images (**bottom**).

**Figure 14:**
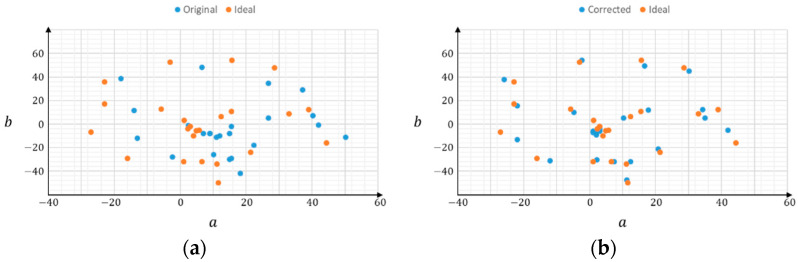
The distribution of 24 colors in ab plane: (**a**) original vs. ideal, and (**b**) corrected vs. ideal colors.

The proposed system was first set to capture an image once every 30 min; thus, we could observe the color correction results at different times under various lighting conditions. From our experiences in farming fields, the correction results were almost the same as what we saw. Figure 12 shows some results of our color correction method at three timestamps on one of the sites. The upper and bottom rows represent the original and color-corrected images, respectively, and the left, middle, and right columns represent the three different times. It can be observed from this figure that the water color varied with time and lighting conditions. Similarly, Figure 13 shows the color correction results at different times at another experimental site. In addition, from the numerical observations in the CIE Lab color model, the ab values of the corrected color are closer to the ideal value (can be derived from Table 1) than before the color correction. Figure 14 shows an example of the distribution of 24 colors in the ab plane because the a and b values are related to chromaticity, which is the objective quality of a color regardless of its luminance. In subplot (a), the blue dots represent the originally captured color of the 24 color blocks, whereas the orange dots represent the targets of ideal colors. Similarly, in subplot (b), the blue dots represent the corrected colors of the 24 blocks. Evidently, the corrected colors are much closer to the ideal values. Thus, it can be concluded that the proposed color correction method is feasible.

### 3.2. Results of Image Segmentation

**Figure 15:**
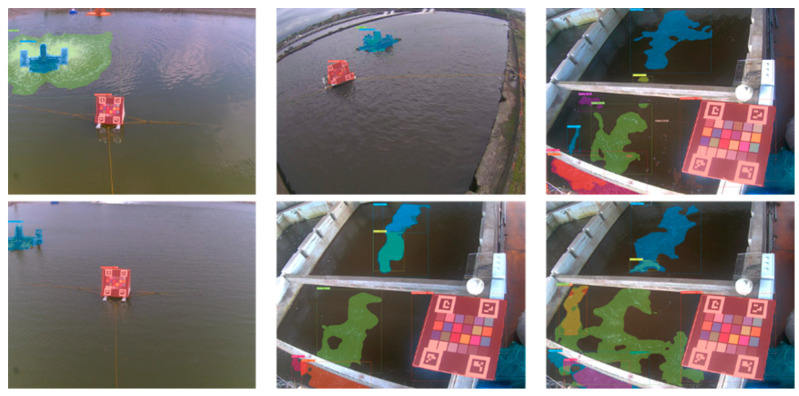
Results of image segmentation from different scenes at three sites.

In the present study, a fully convolutional model, YOLACT++ [32], was implemented for instance segmentation because it is superior to other methods in terms of the balance between time efficiency and accuracy. The choice of model is not the focus of this study because it can be replaced by any semantic or instance segmentation method. Therefore, we only evaluated the performance in different settings of the YOLCAT-based models; thus, the parameters of the model could be determined properly. Table 5 summarizes the quantitative comparison among the six settings of the employed models, where 400, 550, and 700 denote the base image size. The symbol ++ indicates that the improved YOLACT model, namely YOLCAT++, was used. Here, we tested the performance based on the computation time, FPS, AP50, and AP75 indices using our own collected images from the experimental sites. It can be seen that the improved YOLACT model with a ResNet-200 backbone (denoted in bold font) outperformed the others when the average precision was considered. Therefore, we selected this model as the default in this study because its FPS index also met the requirement. Figure 15 shows the results of segmentation for different scenes at our experimental sites, including indoor and outdoor cases.

### 3.3. Results of Color Identification

The final result of the proposed system is the representative color of the monitored pond. In this subsection, the detailed implementation of training a color identification model is introduced; thus, the results of model inference are provided later. We also collaborated with experts in aquaculture fields to provide us with correct ground truths for classification labeling. For any captured image, the candidate patches were extracted and identified. In the detailed implementation, we first cropped N candidates for water patches using the aforementioned fuzzy inference system. Subsequently, the $N_{1}$ candidates with the highest degree values were selected. In this study, N=11 and $N_{1}=5$ were predetermined based on the on-site experiences. As shown in Figure 11, the first half of the network is a ResNet-50 feature extractor, whose input is a normalized image size of 224 × 224 pixels and a feature vector output of 2048 × 1. The second half is a fully connected neural network applied to conduct 19-class classification. Their complete compositions are listed in Table 6 and Table 7.

**Figure 16:**
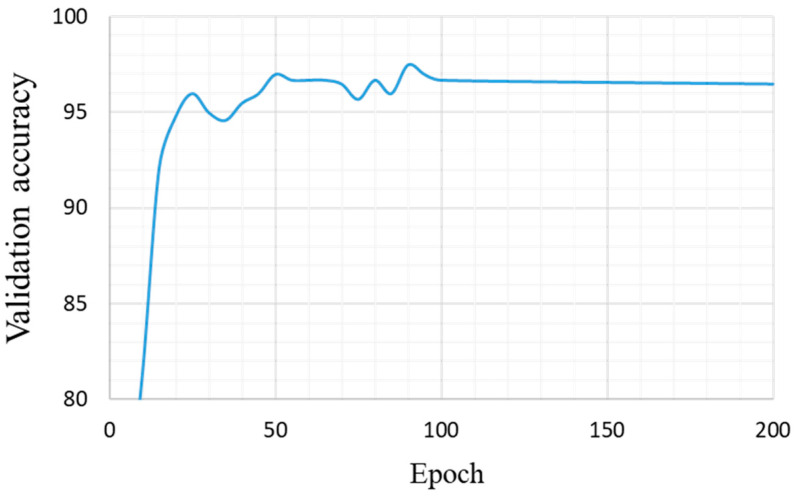
Validation accuracy per epoch.

During this experiment, we collected 9500 samples to form our training dataset, and 1900 samples for testing. Because of the difficulty in gathering sufficient numbers of real samples of various colors, a part of the dataset was generated by data augmentation and synthesis techniques. The samples were manually classified into 19 classes of water color, as listed in Table 4. Notably, there is another class, known as unknown, referring to other colors that cannot be classified into these 19 categories. We set the hyperparameters as follows: maximum epoch of 200; dropout probability of 0.5; batch size of 32; optimized using Adam with commonly used settings of $\beta_{1}=0.9$, $\beta_{2}=0.999$, and $\epsilon =10^{-8}$; and the learning rate $\eta =10^{-4}$. Figure 16 shows the per-epoch trend of the validation accuracy, which reached a maximum of 97.5% at epoch 85. This plot is helpful for observing overfitting. Therefore, we selected the model trained at epoch 85 as the final model for color identification. Table 8 lists the confusion matrix for the testing samples, where a value of 0 was preserved by a blank. The overall accuracy of the test data was 96.9%. As observed from this table, class 12, namely tawny (yellowish-brown), had the lowest accuracy of 88% because some tawny samples were classified as dark brown or dark yellow (Table 9).

## 4. Discussions

### 4.1. Representative Color Determination

After the water color identification stage, the proposed system provided $N_{1}=5$ water-only image patches and their classified color categories. If more than half of these five classified colors are the same, the color is selected as being the most representative. Otherwise, the color that appears most frequently is selected. If no color appears more frequently than the others, the color identified with the highest confidence score is suggested. These determination criteria can be adjusted in practice according to actual situations.

### 4.2. Overall System Construction on Site

The key methodologies of the proposed system have been described in the previous sections. Here, we discuss the deployment of the proposed system at an experimental site.

**Figure 17:**
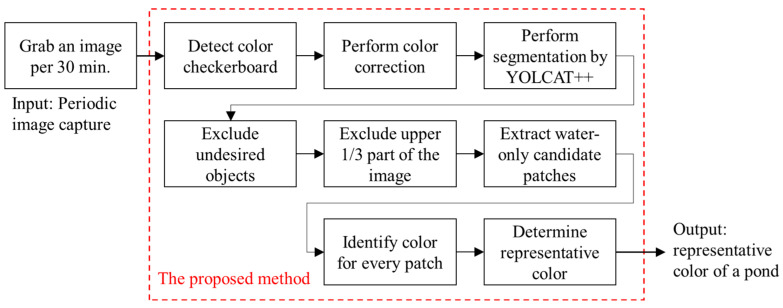
Block diagram of the proposed system implemented at experimental sites.

As shown in Figure 2, a color checkerboard was first placed on the water. The normal vector of the checkerboard was recommended to be parallel to the north–south line according to our experiences to reduce backlight interference during daylight hours. Consequently, the camera position can be easily determined based on the selected position of the checkerboard. In our experiments, a proper image included the water area and checkerboard captured from a suitable camera position. After the system was set up, the water color identification algorithm was executed. Figure 17 shows the block diagram implemented in this study. The proposed system recorded an image every 30 min. Subsequently, it identified the color of the water in the monitored pond and stored the resultant data on the cloud. During the growth of organisms, the trend of the changes in the color of the water served as an important indicator for aquaculture management.

### 4.3. Further Discussion on Proposed FIS

**Figure 18:**
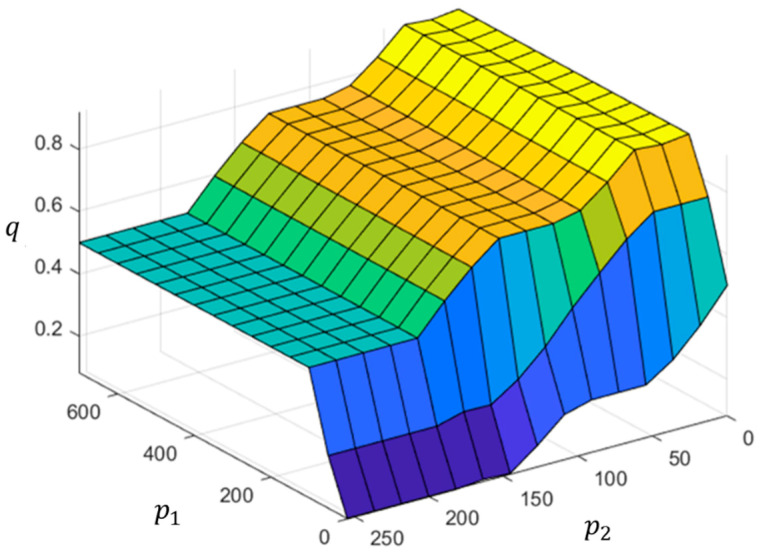
Input–output mapping surface of the proposed FIS.

In Section 2.2.2, an FIS was presented to derive the degree to which an extracted patch could be considered as a candidate water-only patch. When an input pair enters the FIS, several steps are required to compute the crisp output, including fuzzification, firing rules, inferencing, and defuzzification. To reduce the computation time, we transformed the proposed FIS into an input–output mapping, i.e., $q=\phi \left(p_{1},p_{2}\right)$, which can be precalculated and constructed using a lookup table for $p_{1}=1,2,\ldots ,L_{1}$ and $p_{2}=1,2,\ldots ,255$. Figure 18 shows the predetermined mapping surface, in which the horizontal plane is formed by the lines $p_{1}$ and $p_{2}$ as axes, and the vertical axis is the crisp output of q. Accordingly, the computation time of the proposed FIS is reduced significantly using the lookup table.

## 5. Conclusions

In this work, we have presented an identification system and an algorithm for monitoring the color of water in aquaculture ponds. The proposed system primarily comprises a camera, a color checkerboard, and a cloud computing platform, whereas the algorithm comprises a sequence of stages in which color correction, image segmentation, water-only patch extraction, and color identification of patches are successively performed. The effectiveness of color correction using our own checkerboard was verified under different lighting conditions. We then applied instance segmentation followed by a fuzzy inference system to extract candidate water-only image patches. Finally, a color identification model based on deep learning was used to determine the representative color of the monitored aquaculture pond. The output of the proposed system can be used directly as a periodic log report to the FA-COA, Taiwan.

In this study, we have mainly considered a vision-based system designed to monitor the color of water in an aquaculture pond. Therefore, the results of color identification are consistent and do not differ individually. Moreover, the proposed system provides the functionalities of uploading log reports and recording all historical data. Based on these recorded data, farmers or managers of aquaculture fields can make farming decisions accurately. Some important issues remain to be investigated, such as replacing traditional water quality sensors with remote sensors. We are also evaluating the feasibility of using a camera to measure water quality. The changing trend of the color of the water could be used to estimate whether water quality is deteriorating if an indicator could be defined in terms of vision-based recognition. We intend to continue pursuing this research at more experimental sites.

## Figures and Tables

**Table 1:** The 24 colors used in our color checkerboard.

Color	[R,G,B]	Color	[R,G,B]	Color	[R,G,B]	Color	[R,G,B]
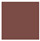	[111,77,71]	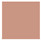	[189,149,132]	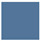	[93,116,151]	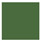	[86,106,67]
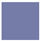	[118,123,164]	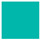	[92,181,170]	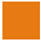	[206,121,37]	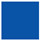	[50,85,164]
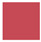	[172,75,85]	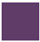	[88,62,103]	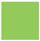	[164,201,100]	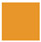	[212,149,55]
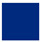	[20,46,123]	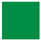	[67,140,76]	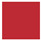	[162,46,55]	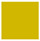	[205,185,0]
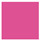	[194,83,145]	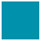	[47,144,170]	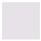	[229,225,230]	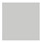	[201,201,200]
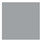	[158,161,162]	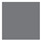	[118,119,123]	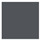	[83,86,90]	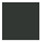	[55,58,54]

**Table 2:** Parameters of fuzzy sets of antecedent variables.

$\mathrm{Variable}\;p_{1}$	$\alpha_{1}^{1}$	$\alpha_{1}^{2}$	$\alpha_{1}^{3}$	$\alpha_{1}^{4}$	$\alpha_{1}^{5}$
	0	25	50	75	100
$\mathrm{Variable}\;p_{2}$	$\alpha_{2}^{1}$	$\alpha_{2}^{2}$	$\alpha_{2}^{3}$	$\alpha_{2}^{4}$	$\alpha_{2}^{5}$
	0	45	90	135	180

**Table 3:** Fuzzy rule table for determining the degree to which a specified crop is selected as a candidate patch.

	$p_{1}$	VS	S	M	L	VL
$p_{2}$						
VS		M	L	L	VL	VL
S		S	M	L	L	VL
M		S	S	M	L	L
L		VS	S	S	M	L
VL		VS	VS	S	S	M

**Table 4:** Coding status of 19 water color categories.

Index	Major Color	Major Code	Sub Color	Sub Code	Full Code
1	Green	001	Blue-green	001	001-001
2			Dark green	002	001-002
3			Green	003	001-003
4			Light green	004	001-004
5	Brown	002	Dark brown	001	002-001
6			Brown	002	002-002
7			Light brown	003	002-003
8	Red	003	Dark red	001	003-001
9			Pink	002	003-002
10			Light red	003	003-003
11			Red	004	003-004
12	Yellow	004	Tawny	001	004-001
13			Dark yellow	002	004-002
14			Light yellow	003	004-003
15			Yellow	004	004-004
16	Dark	005	Dark gray	001	005-001
17			Dark	002	005-002
18			Gray	003	005-003
19	Blue	006	Blue	001	006-001

**Table 5:** The performance comparison among different settings of YOLCAT-based models.

Model	Backbone	Time (ms)	FPS	AP_50_	AP_75_
YOLACT-400	ResNet-101	18.34	54..5	40.8	23.4
YOLACT-550	ResNet-101	24.51	40.8	47.3	29.8
YOLACT-700	ResNet-101	33.77	29.6	49.3	30.6
YOLACT-550++	ResNet-101	28.63	34.9	52.4	34.9
YOLACT-700++	ResNet-101	36.57	27.3	54.1	36.5
YOLACT-700++	ResNet-200	42.02	23.8	56.7	38.4

**Table 6:** The architecture of the feature extractor in our color identification model.

Feature Extractor: ResNet-50 Encoder				
Layer Name	Kernel Size	Stride	Channels	Repeat Times
Convolution	7×7	2	3→64	1
Pooling	3×3	2	−	1
Res-Block 1	$\left[\begin{array}{c} 1\times 1\\ 3\times 3\\ 1\times 1 \end{array}\right]$	1	64→256	3
Res-Block 2	$\left[\begin{array}{c} 1\times 1\\ 3\times 3\\ 1\times 1 \end{array}\right]$	1	256→512	4
Res-Block 3	$\left[\begin{array}{c} 1\times 1\\ 3\times 3\\ 1\times 1 \end{array}\right]$	1	256→1024	6
Res-Block 4	$\left[\begin{array}{c} 1\times 1\\ 3\times 3\\ 1\times 1 \end{array}\right]$	1	1024→2048	3

**Table 7:** The architecture of a fully connected network in our color identification model.

Classifier: Fully Connected Neural Network		
Layer Name	Input Dimension	Output Dimension
FC-1 ^1^	2048	1000
FC-2 ^1^	1000	100
FC-3 ^1^	100	19
Softmax ^2^	19	19

^1^ FC = Fully connected layer. ^2^ Softmax was used to map the output of a neural network to a probability distribution over the predicted output classes. This ensures that the sum of all the output elements equals 1.

**Table 8:** Confusion matrix of the color identification using testing samples.

		**Predicted Class**																		
		1	2	3	4	5	6	7	8	9	10	11	12	13	14	15	16	17	18	19
**True Class**	1	0.99															0.01			
	2		1.0																	
	3			0.94	0.06															
	4				1.0															
	5					0.9			0.05				0.05							
	6						0.94	0.05			0.01									
	7							0.99			0.01									
	8					0.04			0.96											
	9									1.0										
	10							0.01			0.99									
	11										0.02	0.98								
	12					0.04							0.88	0.08						
	13												0.08	0.91			0.01			
	14														1.0					
	15														0.04	0.96				
	16		0.01			0.02							0.01				0.96			
	17																	1.0		
	18																		1.0	
	19																			1.0

**Table 9:** Misclassified cases for class 12: tawny color.

Patch sample	True Class	Predicted Class
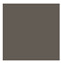	Tawny	Dark brown
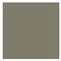	Tawny	Dark yellow

## Data Availability

The data used to support the findings of this study are available from the corresponding author upon request.
